# A third SARS-CoV-2 spike vaccination improves neutralization of variants-of-concern

**DOI:** 10.1038/s41541-021-00411-7

**Published:** 2021-12-03

**Authors:** Mitch Brinkkemper, Philip J. M. Brouwer, Pauline Maisonnasse, Marloes Grobben, Tom G. Caniels, Meliawati Poniman, Judith A. Burger, Ilja Bontjer, Melissa Oomen, Joey H. Bouhuijs, Cynthia A. van der Linden, Julien Villaudy, Yme U. van der Velden, Kwinten Sliepen, Marit J. van Gils, Roger Le Grand, Rogier W. Sanders

**Affiliations:** 1 Department of Medical Microbiology, Amsterdam UMC, University of Amsterdam, Amsterdam Infection & Immunity Institute, Amsterdam, 1105 AZ The Netherlands; 2 Center for Immunology of Viral, Auto-immune, Hematological and Bacterial diseases (IMVA-HB/IDMIT), Université Paris-Saclay, Inserm, CEA, Fontenay-aux-Roses, France; 3 AIMM Therapeutics BV, Amsterdam, 1105 BA The Netherlands; 4 J&S Preclinical Solutions, Oss, 5345 RR The Netherlands; 5 Department of Microbiology and Immunology, Weill Medical College of Cornell University, New York, NY USA

**Keywords:** SARS-CoV-2, Protein vaccines, Protein vaccines

## Abstract

The emergence of SARS-CoV-2 variants that are more resistant to antibody-mediated neutralization pose a new hurdle in combating the COVID-19 pandemic. Although vaccines based on the original Wuhan sequence have been shown to be effective at preventing COVID-19, their efficacy is likely to be decreased against more neutralization-resistant variants-of-concern (VOC), in particular, the Beta variant originating in South Africa. We assessed, in mice, rabbits, and non-human primates, whether a third vaccination with experimental Wuhan-based Spike vaccines could alleviate this problem. Our data show that a third immunization improves neutralizing antibody titers against the variants-of-concern, Alpha (B.1.1.7), Beta (B.1.351), Gamma (P.1), and Delta (B.1.617.2). After three vaccinations, the level of neutralization against Beta was similar to the level of neutralization against the original strain after two vaccinations, suggesting that simply providing a third immunization could nullify the reduced activity of current vaccines against VOC.

## Main text

The coronavirus disease 2019 (COVID-19) pandemic caused by severe acute respiratory syndrome coronavirus 2 (SARS-CoV-2) remains to present a major burden on society, with more than 250 million people infected since the start of the pandemic and around 5 million SARS-CoV-2 related deaths as of November 1, 2021 (https://covid19.who.int/). The main component of vaccines is the SARS-CoV-2 spike (S), which elicits neutralizing antibody (NAb) responses that protect against infection^1^. These NAbs mainly target the receptor-binding domain (RBD) or the N-terminal domain (NTD) of the S protein. The U.S. Food and Drug Administration (FDA), European Medicines Agency (EMA), and other regulatory agencies have approved several effective vaccines containing S based on the virus lineage that originated in Wuhan^2–5^ and multiple vaccines are now being rolled out in large vaccination campaigns^6–9^. However, SARS-CoV-2 variants-of-concern (VOC) have emerged that are less sensitive to neutralization by plasma of convalescent patients or vaccinated individuals^10–14^.

While SARS-CoV-2 was spreading to Europe and the Americas, the B.1 lineage quickly became the dominant strain. The B.1 lineage carries the D614G substitution which enhances viral replication but slightly increases susceptibility to neutralization^15,16^. The Alpha VOC, first identified in the United Kingdom carries deletions at positions 69-70 and 144 in the NTD and a N501Y mutation in the RBD. The Beta and Gamma variants, which originated in South Africa and Brazil, respectively, carry the K417N/T, E484K, and N501Y amino acid changes in the RBD. The Delta variant, which originated in India, carries the T19R, G142D, 156–157 deletion and R158G in the NTD, and L452R and T478K in the RBD^17^. While the Alpha variant is more infectious, it is only slightly more resistant to neutralization by sera from convalescent patients and vaccinees compared to B.1^14,15,18–21^. In contrast, mutations in the Beta S decrease the neutralization sensitivity of convalescent and vaccine sera by approximately sixfold (range: 4–42)^10–14,21^, while mutations in Gamma decrease neutralizing activity by approximately threefold (range: 2–7)^10,12,21^. Delta shows increased transmissibility^22^ and was approximately threefold less sensitive to neutralization (range: 2–5)^23,24^. This decreased potency might pose a threat towards controlling the SARS-CoV-2 pandemic by vaccination. Indeed, several vaccines were shown to be less effective against these VOC in phase 2 and 3 studies^9,25–28^. For example, the AstraZeneca vaccine showed reduced efficacy against Alpha and was virtually ineffective at preventing virus infection in a trial in South Africa where the Beta VOC dominated during the trial^26,27^.

**Fig. 1:**
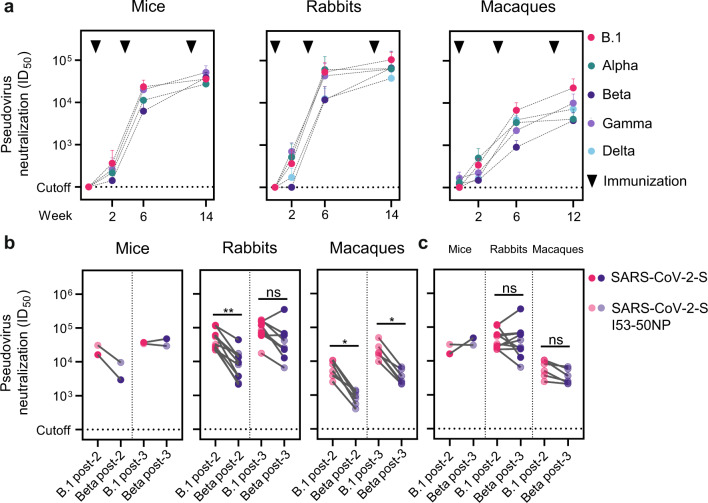
Neutralizing antibody responses against SARS-CoV-2 VOC in mice, rabbits, and cynomolgus macaques after one, two, and three vaccinations with Wuhan-based Spike vaccines. **a** Longitudinal analysis of pseudovirus neutralization of B.1, and VOC Alpha, Beta, and Gamma in mice (left), and of B.1, Alpha, Beta, Gamma, and Delta in rabbits (middle) and macaques (right). Datapoints represent mean ID_50_’s, error bars indicate SD. **b** Paired comparisons of B.1 and Beta neutralization 2 weeks after the second and third vaccination in mice (left), rabbits (middle), and macaques (right). **c** Paired comparisons of Beta neutralization 2 weeks after the third vaccination and B.1. neutralization after the second vaccination in mice (left), rabbits (middle), and macaques (right). Mice and rabbits groups immunized with SARS-CoV-2 S-I53-50NP and SARS-CoV-2 S were combined for the analyses because the responses were overall quite comparable, but the individual data are given in Supplementary Tables 1 and 2. Mouse sera were pooled per group because of low serum amounts. The neutralization titers between variants were compared using the Wilcoxon test (*P < 0.05; **P < 0.01).

Here, we assessed whether a third vaccination with experimental Wuhan-based Spike vaccines could mitigate the reduced neutralization activity against the Alpha, Beta, Gamma, and Delta VOC. First, we subcutaneously immunized five BALB/c mice three times at weeks 0, 4, and 12 with 10 µg SARS-CoV-2 S soluble protein adjuvanted in polyinosinic-polycytidylic acid (poly-IC). Second, we intramuscularly immunized five New Zealand White rabbits three times at weeks 0, 4, and 12 with 30 µg SARS-CoV-2 S soluble protein adjuvanted in squalene emulsion. Furthermore, we reanalyzed mouse and rabbit samples from previously published vaccination studies in which we investigated a two-component SARS-CoV-2 S-I53-50 nanoparticle (NP) vaccine and used the same study design^29^. As we only observed small differences in pseudovirus neutralization between the SARS-CoV-2 S and SARS-COV-2 S-I53-50NP groups, we combined the animals from both groups in our analyses in Fig. 1. Finally, we reanalyzed the sera from six cynomolgus macaques that were immunized intramuscularly three times at weeks 0, 4, and 10 with 50 µg of SARS-CoV-2 S-I53-50NPs adjuvanted in MPLA liposomes^29^. NAb responses were analyzed 2 weeks after each immunization. Mouse sera were pooled per group and timepoint because the serum volumes were too limited to test the individual sera against all VOC. Neutralization activity was measured using a pseudovirus-based assay, of which the results correlated very well with those obtained with authentic virus-neutralization assays^29,30^.

In all animal models tested, neutralization activity against B.1, Alpha, Beta, and Delta was already detectable 2 weeks after the first immunization, and in most individual animals (mouse sera were not tested for neutralizing activity against Delta due to limiting serum amounts). Thus, 7/10 rabbits and 5/6 macaques neutralized Alpha at week 2, 9/10 rabbits and 5/6 macaques neutralized Gamma, and 4/10 rabbits and 5/6 macaques neutralized Delta at that timepoint with a titer >100 (Supplementary Tables 2 and 3). In contrast, Beta neutralization was detected in none of the rabbits and 3/6 macaques at week 2 (Supplementary Tables 2 and 3). In mice, neutralization of all lineages was detected in the SARS-CoV-2 S-I53-50NP immunized animals 2 weeks after the first immunization, but not in the SARS-CoV-2 S immunized group (Supplementary Table 1). Neutralization of the B.1 lineage reached a titer of 628, while the titers against Alpha and Gamma were twofold lower (titers of 331 and 419, respectively), and against Beta was fourfold lower (titer of 183). In rabbits, the neutralization titers against B.1 reached a mean titer of 365 2 weeks after the priming vaccination, while the titers against Alpha and Gamma were actually slightly higher (mean titers of 519 and 703, respectively) and the titer against Delta was twofold lower (mean titer of 171). In macaques, one immunization induced mean neutralization titers of 337 and 497 against B.1 and Alpha, respectively, while the mean neutralization titer against Gamma was slightly lower (mean titer of 223) and the titers against Beta and Delta were close to the background (mean titers of 148 and 166, respectively). Overall, we conclude that one immunization with Wuhan-based S vaccines induced considerably more frequent and stronger neutralizing responses against B.1, Alpha, Gamma, and Delta than against Beta, with some differences between species.

In mice, the second immunization increased the mean neutralizing activity against B.1 to 23,422 and the neutralizing titer against Gamma was similar (mean titer of 20,055), while the titers against Alpha and Beta were twofold and fourfold lower, respectively (mean titers of 11,198 and 6,240, respectively) (Fig. 1a, b, left). When assessing the individual groups, a threefold difference in Beta neutralizing titer was observed between the SARS-CoV-2 S and SARS-CoV-2 S-I53-50NP groups (titers of 2,943 and 9,537, respectively). After the third immunization, neutralization activity against B.1 and the three VOC was similar and strong (means titers of 35,746 for B.1; 27,375 for Alpha; 38,592 for Beta; and 51,426 for Gamma) (Fig. 1a, b, left).

The second immunization led to high neutralizing titers in rabbits and these were similar for B.1, Alpha, and Gamma (mean titers of 53,396, 60,731, and 43,535, respectively), while the neutralizing titers against Beta and Delta were five- and fourfold lower compared to B.1 (mean titer of 11,774, *P* = 0.002; 12,412 *P* = 0.002, respectively) (Fig. 1a, b, middle). The third immunization increased the mean neutralizing titer against B.1 to 104,654, and the neutralizing titers against Alpha, Beta and Gamma were close to twofold lower, but not significantly different from that against B.1 (mean titers of 64,116, 66,723 and 63,178, respectively) (Fig. 1a, b, middle), similar to what we observed in mice. The neutralizing titer against Delta was threefold lower compared to that against B.1 (mean titer of 37,941, *P* = 0.002) (Fig. 1a, middle). The neutralization titers after three vaccinations were significantly higher for B.1, Beta, and Delta compared to two vaccinations (mean titers of 53,396 vs 104,654, *P* = 0.0059 for B.1; 60,731 vs 64,116, not significant for Alpha; 11,774 vs 66,723, *P* = 0.0039 for Beta; 43,535 vs 63,178, not significant for Gamma; 12,412 vs 37,941, *P* = 0.0059 for Delta).

**Fig. 2:**
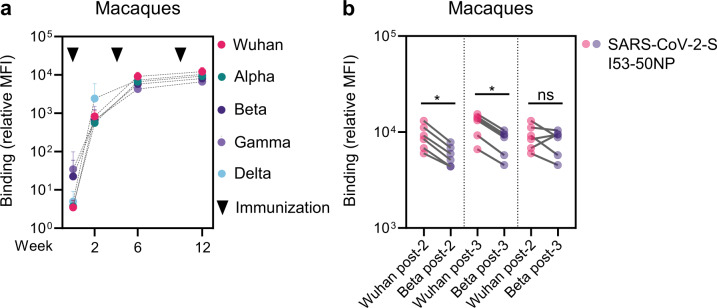
Antibody binding responses against VOC in cynomolgus macaques after one two and three vaccinations with Wuhan-based Spike vaccine. **a** Longitudinal analysis of Ab binding to Wuhan S and VOC Alpha, Beta, Gamma, and Delta in macaques. Datapoints represent mean relative MFIs, error bars indicate SD. **b** Paired comparisons of Ab binding to Wuhan S and Beta S two weeks after the second and third vaccination, and to Wuhan S two weeks after the second vaccination and Beta S two weeks after the third vaccination. The relative MFIs between variants were compared using the Wilcoxon test (*P < 0.05).

The results in macaques were slightly different than those obtained in mice and rabbits. Thus, 2 weeks after the first boost, at week 6, neutralization activity against B.1 reached a mean titer of 6,734, while titers were two-, eight-, three- and twofold lower against Alpha (mean titer of 3,436, not significant), Beta (mean titer of 897, *P* = 0.0313), Gamma (mean titer of 2,220, *P* = 0.0313) and Delta (mean titer of 3,967, not significant), respectively (Fig. 1a, b, right). After the third immunization, neutralization against B.1 reached a mean titer of 22,732. The titers against Alpha, Beta, Gamma and Delta were five-, six-, two- and threefold lower compared that against B.1 at week 12 (mean titers of 4,154, *P* = 0.0313; 3,850, *P* = 0.0313; 9,927, not significant; 7,180, *P* = 0.0313, respectively) (Fig. 1a, b, right). The neutralization titers after three vaccinations in macaques were significantly higher for B.1, Beta, and Gamma compared to two vaccinations (mean titers of 6,734 vs 22,732, *P* = 0.0313 for B.1; 3,436 vs 4,154, not significant for Alpha; 897 vs 3,850, *P* = 0.0313 for Beta; 2,220 vs 9,927, *P* = 0.0313 for Gamma; 3,967 vs 7,180, not significant for Delta). The neutralization titers against B.1 and Beta were significantly higher after three vaccinations in both rabbits and macaques compared to the titers after the second dose. Interestingly, in the rabbit sera, the neutralization titer against Delta was the lowest of all the VOC, while in macaques this was not the case. To assess whether the decrease in neutralization potency of the VOC could be explained by a decreased Ab binding, we conducted a custom Luminex-bead-based serological assay. Ab binding in macaque serum samples to S from all VOC was compared to binding to the original Wuhan S. Ab binding over time shows less variation between the different variants compared to neutralization (Fig. 2a). Considering that S form different variants share a lot of the same non-neutralizing epitopes this could be expected. However, both at weeks 6 and 12, Ab binding to Alpha, Beta, Gamma, and Delta S was significantly lower than binding to Wuhan S, as shown for Beta in Fig. 2b. Ab binding relative MFIs showed correlation with neutralization titers at week 12 (Spearman *r* = 0.6147, *P* = 0.0003) (Supplementary Fig. 1).

Because two vaccinations with Wuhan-based S vaccines are highly protective against B.1 in large-scale efficacy trials, we compared the level of neutralization against Beta, the VOC with most neutralization resistance based on literature and our experiments, after three immunizations, with that against B.1 after two immunizations. In all three animal models, the neutralization titers against Beta were higher or similar after three immunizations compared to the B.1 neutralizing titers after two immunizations (Fig. 1c). The same pattern was observed for Ab binding in macaques (Fig. 2b). Overall, these data show that the antibody-mediated neutralization of VOC, in particular Beta, can be improved by a third immunization with a SARS-CoV-2 vaccine based on the Wuhan S sequence.

Both Moderna and Pfizer-BioNTech have announced that they are working on variant-targeting booster shots to deal with the more neutralization-resistant SARS-CoV-2 lineages. Moderna has published preliminary data of a phase 2 clinical study in which participants that previously received two doses of the mRNA-1273 were boosted with mRNA-1273 or mRNA-1273.351, which is specifically targeted against the Beta VOC^31^. Neutralization of Beta reached peak NAb titers similar to the neutralization of B.1 after two doses in both groups, similar to what we show in macaques immunized with our subunit vaccine. They reported a 1.6-fold increase in pseudovirus neutralization against Beta (geometric mean titer ID_50_ of 1,400 versus 864) in the mRNA-1273.351 boosted group. Whether this modest difference will result in improved protection against Beta remains to be seen, but the overall results from that study suggest that the third immunization with a SARS-CoV-2 vaccine based on the original Wuhan sequence could provide significant protection against the current VOC, in line with the findings from our study. While research into variant-targeting vaccines is important, another pragmatic solution to protecting against VOC is simply a booster vaccination with the currently available Wuhan sequence-based vaccines. The surplus of vaccines that many countries have procured could be used for this purpose. The timing of such boost vaccination will be important, as immunizing too quickly after the second immunization might be counterproductive, similar to what has been observed for shortly spaced vaccinations in individuals who previously experienced COVID-19 infection^32^. Furthermore, whether an individual experienced COVID-19 prior to vaccination should be factored in^33,34^.

## Methods

### Cell lines

All experimental models were described before^29^. The HEK293F (Life Technologies) and HEK293T (ATCC CRL-11268) cell lines are human embryonic kidney cells transformed to have increased recombinant protein production and increased retrovirus production. HEK293T cells were cultured in DMEM supplemented with 10% fetal bovine serum (FBS), streptomycin (100 μg/mL) and penicillin (100 U/mL) at 37 °C with 5% CO_2_ in flasks. HEK293F cells were cultured in 293FreeStyle expression medium (Life Technologies) at 37 °C with 8% CO_2_ and shaking at 125 rpm. HEK293T/ACE2 cells are transformed to express human angiotensin-converting enzyme 2. The HEK293T/ACE2 cells were cultured in DMEM supplemented with 10% FBS, streptomycin (100 μg/mL) and penicillin (100 U/mL) at 37 °C with 5% CO_2_ in flasks.

### Mice

Female BALB/cAnNCrl mice that were 8 weeks of age were ordered from Charles River Laboratories and housed at the Animal Research Institute Amsterdam under BSL-2 conditions. All procedures were done in accordance with the Dutch Experiment on Animals Act and were approved by the Animal Ethics Committee of the Amsterdam UMC (Permit number 17-4045).

### Rabbits

For the rabbit study, Female New Zealand White rabbits from multiple litters of 2.5–3 kg were used. Immunizations were performed under permits with approval number C0084-20 at Covance Research Products, Inc. (Denver, PA, USA). All procedures complied with all relevant ethical regulations and protocols of the Covance Institutional Animal Care and Use Committee.

### Cynomolgus macaques

Female cynomolgus macaques aged 56-66 months that originated from Mauritian AAALAC certified breeding centers were used in this study. The animals were housed under BSL-2 and BSL-3 containment when necessary (Animal facility authorization #D92-032-02, Préfecture des Hauts de Seine, France) in IDMIT infrastructure facilities (CEA, Fontenay-aux-roses). All in compliance with the French regulations and the Standards for Human Care and Use of Laboratory Animals, European Directive 2010/63/EU, of the Office for Laboratory Animal Welfare (OLAW, assurance number #A5826-01, US). The study was authorized by the “Research, Innovation and Education Ministry” under registration number APAFIS#24434-2020030216532863v1. Study protocols were approved by the institutional ethical committee “Comité d’Ethique en Expérimentation Animale du Commissariat à l’Energie Atomique et aux Energies Alternatives” (CEtEA #44) under statement number A20-011.

### Construct design

The creation of the SARS-CoV-2-S-Foldon encoding pPPI4 plasmid was previously described^30^. In brief, a gene encoding residues 1-1138 of SARS-CoV-2 S (WuhanHu-1; GenBank: MN908947.3) was cloned into a pPPI4 backbone containing a T4 trimerization domain followed by a hexahistidine-tag with PstI-BamHI digestion and ligation. This construct contains proline substitutions at 986 and 987, and a GGGG substitution at the furin cleavage site (682–685). The SARS-CoV-2-S-B.1_Δ19_, SARS-CoV-2-S-B.1.1.7_Δ19_, SARS-CoV-2-S-B.1.351_Δ19_, SARS-CoV-2-S-P.1_Δ19_ plasmids were described elsewhere and SARS-CoV-2-S-B.1.617.2_Δ19_ was made as described elsewhere^21^. In brief, gBlock gene fragments (Integrated DNA technologies) we ordered and cloned SacI/ApaI into the pCR3 SARS-CoV-2-S_Δ19_ expression plasmid (GenBank: MT449663.1).

### Protein production

HEK293F cells (Invitrogen) were transiently transfected with SARS-CoV-2 S-Foldon pPPI4. Cells were grown in Freestyle medium (Life Technologies) and transfected at a density of 0.8–1.2 million cells/mL. A mixture of PEImax (1 µg/mL) and expression plasmid (312.5 µg/mL) in OptiMEM (GIBCO) was made on the day of transfection and added to the cells. After 6 days, cells and growth medium were transferred into centrifuge buckets and spun down at 3000 × *g* for 30 min. Supernatants were filtered through 0.22-µm Steritop filters (Merck Millipore). After filtration, spikes were purified using Ni-NTA agarose beads. Eluted proteins were concentrated and the buffer was changed to PBS using 100 kDa cutoff Vivaspin filters (GE Healthcare). Next, proteins were applied to a Superose 6 increase 10/300 GL column linked to a NGC chromatography system (BIO-RAD) in PBS. The appropriate size-exclusion fractions were pooled and proteins were stored at −80 °C.

### Immunization studies

Balb/cAnNCrl mice were randomly divided into two groups of 5 animals each. Animals received subcutaneous immunizations into the neck skin-fold at weeks 0, 4, and 12. The immunization mixture contained 10 µg of SARS-CoV-2 S or 13 µg of SARS-CoV-2 S-I53-50NPs (equal to 10 mg of SARS-CoV-2 S) adjuvanted with 50 mg of polyinosinic-polycytidylic acid (Poly-IC; Invivogen) diluted in PBS. Blood was collected at weeks -1, 2, 6, and 14. Due to low amounts of sera, the serum samples from animals in both groups were pooled at each timepoint.

Female New Zealand White rabbits were randomly assigned into two groups of five animals each. Animals received intramuscular immunizations at weeks 0, 4, and 12, one in each quadricep. Animals were given 30 µg of SARS-CoV-2 S or 39 µg of SARS-CoV-2 S-I53-50NPs (equal to 30 µg of SARS-CoV-2 S) diluted in PBS formulated 1:1 in Squalene Emulsion adjuvant (Polymun, Klosterneuburg, Austria). The rabbits were bled at weeks 0, 2, 4, 6, and 14.

Macaque study was described in detail before^29^. In brief, Six cynomolgus macaques were immunized with 50 µg of SARS-CoV-2 S-I53-50NP adjuvanted with 500 mg of MPLA liposomes (Polymun Scientific, Klosterneuburg, Austria) diluted in PBS at weeks 0, 4, and 10. Blood, nasal swabs, and saliva samples were taken at weeks 0, 2, 4, 6, 8, 10, and 12.

### Pseudovirus neutralization assay

SARS-CoV-2 pseudoviruses were made by transfecting HEK293T cells (ATCC, CRL-11268), cultured in DMEM (GIBCO), supplemented with 10% FBS, penicillin (100 U/mL), and streptomycin (100 mg/mL), with a pHIV-1NL43DENV-NanoLuc reporter virus plasmid and a SARS-CoV-2-S-B.1_Δ19_, SARS-CoV-2-S-B.1.1.7_Δ19_, SARS-CoV-2-S-B.1.351_Δ19_, SARS-CoV-2-S-P.1_Δ19_ or SARS-CoV-2-S-B.1.617.2_Δ19_ plasmid. The growth medium was replaced after 8 h. The cell supernatant containing the pseudoviruses was harvested 48 h post transfection. The supernatant was centrifuged for 5 min at 500 × *g* and sterile filtered through a 0.22-mm pore size PVDF syringe filter. Pseudoviruses were stored at −80 °C.

For the neutralization assays, HEK293T expressing the SARS-CoV-2 receptor ACE2 (HEK293T/ACE2) were cultured in DMEM (GIBCO), supplemented with 10% FBS, penicillin (100 U/mL), and streptomycin (100 mg/mL) and with GlutaMAX (GIBCO). To determine the neutralization activity in serum, HEK293T/ACE2 cells were seeded at a density of 2 × 10^4^ /well in 96-well plates coated with 50 µg/mL poly-l-lysine. Cells were seeded in the culture medium described above. The next day, duplicate serial dilutions of heat-inactivated serum samples were prepared in the same medium used for the cells and mixed 1:1 with pseudovirus. This mixture was incubated at 37 °C for 1 h. After incubation, it was added to the seeded HEK293T/ACE2 cells in a 1:1 ratio with the cell culture medium. 48 h after adding the serum and virus mix, the cells were lysed and lysates were transferred into half area 96-well white microplates (Greiner bio-one). Luciferase activity in the lysates was measured using the Nano-Glo Luciferase Assay System (Promega) with a Glomax system (Turner BioSystems). Relative luminescence units (RLU) were normalized to those from cells infected with the applicable SARS-CoV-2 pseudovirus. Neutralization titers (ID_50_-values) were determined as the serum dilution at which infectivity was inhibited by 50%. ID_50_-values were derived using duplicate titration curves and interpolation using GraphPad Prism v9 software.

### Luminex assay

A custom Luminex assay was used as described previously^29^. In short, prefusion stabilized trimeric spike proteins of all SARS-CoV-2 variants^21^, were produced in HEK293F cells and purified by Ni-NTA chromatography followed by size-exclusion chromatography step. Prefusion stabilized trimeric SARS-CoV-2 B.1.617.2 Spike was provided by Dirk Eggink and Chantal Reusken (National Institute for Public Health and the Environment, the Netherlands). The spike proteins were covalently coupled at a ratio of 75 µg protein to 12,5 million beads to Luminex Magplex beads with a two-step carbodiimide reaction. Serum samples were used at a 1:50,000 dilution. Beads and diluted samples were incubated overnight, followed by detection with goat-anti-monkey IgG-Biotin (Sigma Aldrich) and Streptavidin-PE (ThermoFisher Scientific). Read-out was performed on a Magpix (Luminex). The resulting mean fluorescence intensity (MFI) values are the median of approximately 50 beads per well and were corrected by subtraction of MFI values from buffer and beads-only wells.

### Reporting summary

Further information on research design is available in the Nature Research Reporting Summary linked to this article.

## Supplementary information

Supplementary Information

Reporting Summary

## Data Availability

The data that support the findings of this study are available from the corresponding author upon reasonable request.
