# Supplementary material for: A third SARS-CoV-2 spike vaccination improves neutralization of variants-of-concern
Source: NPJ Vaccines. 2021 Dec 3;6:146. doi: 10.1038/s41541-021-00411-7 (PMC8642392; doi:10.1038/s41541-021-00411-7)
Supplement: Supplementary file 1 — Supplementary Information [file 41541_2021_411_MOESM1_ESM.pdf]

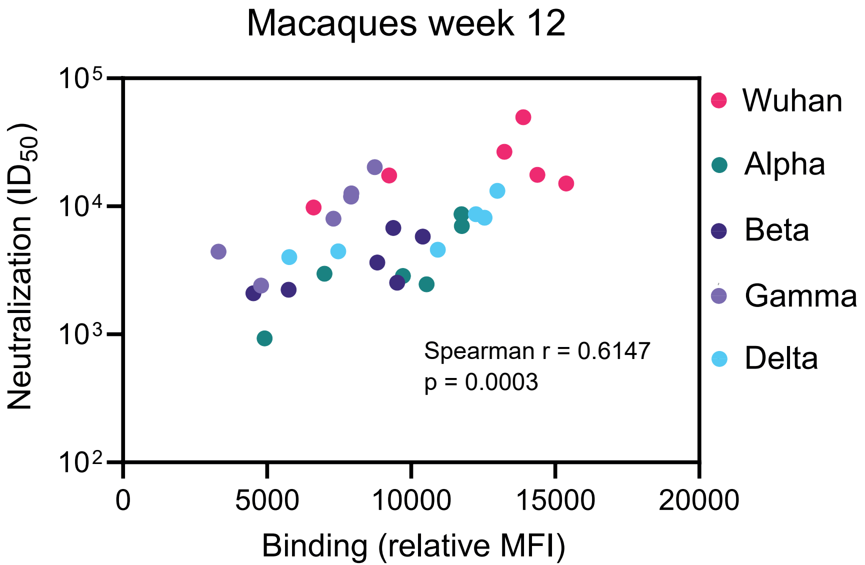

Supplementry Fig. 1: Correlation between antibody binding and neutralizing antibody responses in cynomolgus macaques after 3 vaccinations with Wuhan-based Spike vaccine.
Spearman correlation between Ab binding to Wuhan, Alpha, Beta, Gamma and Delta S and B.1, Alpha, Beta, Gamma and Delta pseudovirus neutralization.

SupplementaryTable 1. SARS-CoV-2 pseudovirus neutralization titers (ID_{50}s) in mice, related to Figure 1.

	Group	SARS-CoV-2-S	SARS-CoV-2-S I53-50NP
	Virus		
Week -1	B.1	<100	<100
	B.1.1.7	<100	<100
	B.1.351	<100	<100
	P.1	<100	<100
Week 2	B.1	<100	628
	B.1.1.7	<100	331
	B.1.351	<100	183
	P.1	<100	419
Week 6	B.1	16163	30680
	B.1.1.7	10181	12214
	B.1.351	2943	9537
	P.1	20591	19519
Week 14	B.1	37410	34081
	B.1.1.7	27741	27009
	B.1.351	47788	29395
	P.1	67013	35838

<100
101-1,000
1,001-10,000
10,001-100,000

Supplementary Table 2: SARS-CoV-2 pseudovirus neutralization titers (ID_{50}s) in rabbits, related to Figure 1.

	Group	SARS-CoV-2-S					SARS-CoV-2-S I53-50NP				
	Animal ID	1	2	3	4	5	6	7	8	9	10
	Virus										
Week 0	B.1	<100	<100	<100	<100	<100	<100	<100	<100	<100	<100
	B.1.1.7	<100	<100	<100	<100	<100	<100	<100	<100	<100	<100
	B.1.351	<100	<100	<100	<100	<100	<100	<100	<100	<100	<100
	P.1	<100	<100	<100	<100	<100	<100	<100	<100	<100	<100
	B.1.617.2	<100	<100	<100	<100	<100	<100	<100	<100	<100	<100
Week 2	B.1	748	498	1293	121	476	<100	<100	<100	<100	<100
	B.1.1.7	1111	315	1002	<100	1612	<100	<100	102	164	589
	B.1.351	<100	<100	<100	<100	<100	<100	<100	<100	<100	<100
	P.1	819	1102	1068	<100	317	285	505	1462	579	794
	B.1.617.2	235	149	394	<100	332	<100	<100	<100	<100	<100
Week 6	B.1	59588	113524	117472	25664	59704	22694	27576	21771	31999	53699
	B.1.1.7	46243	94174	223027	18688	63123	19726	19780	27563	17542	77445
	B.1.351	9346	43931	13414	2134	3671	2320	3673	8021	17370	13860
	P.1	42481	60279	150169	32541	43827	15475	12815	21468	24842	31456
	B.1.617.2	10962	12568	36366	15669	13706	4465	5251	8573	3225	13330
Week 14	B.1	153615	149943	118402	58533	70204	17408	170076	84310	64352	159695
	B.1.1.7	117929	83690	59177	59218	38938	14989	138119	15614	52212	61276
	B.1.351	67176	342660	12755	60371	22586	6510	41368	22952	25415	65433
	P.1	121806	44294	33126	79983	74395	23718	72180	55440	36377	91465
	B.1.617.2	64745	38843	32799	26735	34039	6227	60634	29267	23505	62614

<100
101-1,000
1,001-10,000
10,001-100,000
100,001-1,000,000

Supplementary Table 3: SARS-CoV-2 pseudovirus neutralization titers (ID_{50}s) in cynomolgus macaques, related to Figure 1.

	Group	SARS-CoV-2-S I53-50NP					
	Animal ID	MF5	MF6	MF7	MF8	MF9	MF10
	Virus						
Week 0	B.1	105	<100	<100	<100	<100	<100
	B.1.1.7	135	<100	<100	<100	173	177
	B.1.351	<100	106	104	<100	232	<100
	P.1	<100	136	227	<100	244	201
	B.1.617.2	<100	<100	<100	<100	<100	<100
Week 2	B.1	556	269	288	263	199	457
	B.1.1.7	1056	270	391	<100	592	584
	B.1.351	193	<100	<100	<100	171	222
	P.1	575	129	149	<100	141	243
	B.1.617.2	121	158	294	N.D.	133	122
Week 6	B.1	10611	2464	8101	5087	10403	3736
	B.1.1.7	2298	744	8976	2854	4080	1662
	B.1.351	1262	390	548	801	980	1399
	P.1	1836	213	1678	917	7729	945
	B.1.617.2	3998	962	5860	5531	4048	3458
Week 12	B.1	15062	9805	49810	17401	26728	17583
	B.1.1.7	7012	933	8690	2979	2852	2459
	B.1.351	5789	2100	6798	2237	3638	2535
	P.1	20193	4426	11889	2406	8026	12621
	B.1.617.2	8170	4010	13197	4457	4571	8672

<100
101-1,000
1,001-10,000
10,001-100,000
