# Supplementary material for: A third SARS-CoV-2 spike vaccination improves neutralization of variants-of-concern
Source: NPJ Vaccines. 2021 Dec 3;6:146. doi: 10.1038/s41541-021-00411-7 (PMC8642392; doi:10.1038/s41541-021-00411-7)
Supplement: Supplementary file 2 — Reporting Summary [file 41541_2021_411_MOESM2_ESM.pdf]

nature portfolio

Corresponding author(s): Rogier Sanders

Last updated by author(s): Sep 9, 2021

Reporting Summary

Nature Portfolio wishes to improve the reproducibility of the work that we publish. This form provides structure for consistency and transparency in reporting. For further information on Nature Portfolio policies, see our Editorial Policies and the Editorial Policy Checklist.

Statistics

For all statistical analyses, confirm that the following items are present in the figure legend, table legend, main text, or Methods section.

n/a	Confirmed	
☐	☒	The exact sample size (*n*) for each experimental group/condition, given as a discrete number and unit of measurement
☐	☒	A statement on whether measurements were taken from distinct samples or whether the same sample was measured repeatedly
☐	☒	The statistical test(s) used AND whether they are one- or two-sided<br>*Only common tests should be described solely by name; describe more complex techniques in the Methods section.*
☒	☐	A description of all covariates tested
☒	☐	A description of any assumptions or corrections, such as tests of normality and adjustment for multiple comparisons
☐	☒	A full description of the statistical parameters including central tendency (e.g. means) or other basic estimates (e.g. regression coefficient) AND variation (e.g. standard deviation) or associated estimates of uncertainty (e.g. confidence intervals)
☒	☐	For null hypothesis testing, the test statistic (e.g. *F*, *t*, *r*) with confidence intervals, effect sizes, degrees of freedom and *P* value noted<br>*Give P values as exact values whenever suitable.*
☒	☐	For Bayesian analysis, information on the choice of priors and Markov chain Monte Carlo settings
☒	☐	For hierarchical and complex designs, identification of the appropriate level for tests and full reporting of outcomes
☐	☒	Estimates of effect sizes (e.g. Cohen's *d*, Pearson's *r*), indicating how they were calculated

Our web collection on statistics for biologists contains articles on many of the points above.

Software and code

Policy information about availability of computer code

Data collection	No software was used to collect data
Data analysis	Software used to analyse data is Graphpad Primsm v9

For manuscripts utilizing custom algorithms or software that are central to the research but not yet described in published literature, software must be made available to editors and reviewers. We strongly encourage code deposition in a community repository (e.g. GitHub). See the Nature Portfolio guidelines for submitting code & software for further information.

Data

Policy information about availability of data

All manuscripts must include a data availability statement. This statement should provide the following information, where applicable:

- Accession codes, unique identifiers, or web links for publicly available datasets
- A description of any restrictions on data availability
- For clinical datasets or third party data, please ensure that the statement adheres to our policy

The raw data supporting the findings of the study are available from the corresponding authors upon reasonable request.

Field-specific reporting

Please select the one below that is the best fit for your research. If you are not sure, read the appropriate sections before making your selection.

☒ Life sciences ☐ Behavioural & social sciences ☐ Ecological, evolutionary & environmental sciences

For a reference copy of the document with all sections, see nature.com/documents/nr-reporting-summary-flat.pdf

Life sciences study design

All studies must disclose on these points even when the disclosure is negative.

Sample size	5 mice per group, 5 rabbits per group and 6 macaques per group. These group sizes are similar to what is used in similar studies.
Data exclusions	No data were excluded from analysis
Replication	All measurements were done in duplo. Replications were successful.
Randomization	Animals were assigned to groups randomly
Blinding	Investigators were not blinded.

Reporting for specific materials, systems and methods

We require information from authors about some types of materials, experimental systems and methods used in many studies. Here, indicate whether each material, system or method listed is relevant to your study. If you are not sure if a list item applies to your research, read the appropriate section before selecting a response.

Materials & experimental systems

n/a	Involved in the study	
☐	☒	Antibodies
☐	☒	Eukaryotic cell lines
☒	☐	Palaeontology and archaeology
☐	☒	Animals and other organisms
☒	☐	Human research participants
☒	☐	Clinical data
☒	☐	Dual use research of concern

Methods

n/a	Involved in the study	
☒	☐	ChIP-seq
☒	☐	Flow cytometry
☒	☐	MRI-based neuroimaging

Antibodies

Antibodies used	goat-anti-monkey IgG-Biotin (Sigma Aldrich)
Validation	Antibody binding was validated in house

Eukaryotic cell lines

Policy information about cell lines

Cell line source(s)	HEK293F (Life Technologies) and HEK293T (ATCC CRL-11268)
Authentication	Cell lines were not authenticated
Mycoplasma contamination	Cell lines were tested negative for mycoplasma
Commonly misidentified lines (See ICLAC register)	Non

Animals and other organisms

Policy information about studies involving animals; ARRIVE guidelines recommended for reporting animal research

Laboratory animals	Female BALB/cAnNCrl mice that were 8 weeks of age, Female New Zealand White rabbits from multiple litters of 2.5-3 kg, Female cynomolgus macaques aged 56-66 months

Wild animals	Non
Field-collected samples	Non
Ethics oversight	For mice, All procedures were done in accordance with the Dutch Experiment on Animals Act and were approved by the Animal Ethics Committee of the Amsterdam UMC (Permit number 17-4045). For rabbits, Immunizations were performed under permits with approval number C0084-20 at Covance Research Products, Inc. (Denver, PA, USA). All procedures complied with all relevant ethical regulations and protocols of the Covance Institutional Animal Care and Use Committee. For macaques, All in compliance with the French regulations and the Standards for Human Care and Use of Laboratory Animals, European Directive 2010/63/EU, of the Office for Laboratory Animal Welfare (OLAW, assurance number #A5826-01, US). The study was authorized by the "Research, Innovation and Education Ministry" under registration number APAFIS#24434-2020030216532863v1. Study protocols were approved by the institutional ethical committee "Comité d'Ethique en Expérimentation Animale du Commissariat à l'Energie Atomique et aux Energies Alternatives" (CEtEA #44) under statement number A20-011.

Note that full information on the approval of the study protocol must also be provided in the manuscript.
